# Fast-Evolving Homoplastic Traits Are Best for Species Identification in a Group of Neotropical Wasps

**DOI:** 10.1371/journal.pone.0074837

**Published:** 2013-09-11

**Authors:** Alexander L. Wild, Paul M. Marsh, James B. Whitfield

**Affiliations:** 1 Department of Entomology, University of Illinois at Urbana-Champaign, Urbana, Illinois, United States of America; 2 North Newton, Kansas, USA; Consiglio Nazionale delle Ricerche (CNR), Italy

## Abstract

Biological characters can be employed for both taxonomy and phylogenetics, but is conscripting characters for double duty a good idea? We explore the evolution of characters designed for taxonomic diagnosis in Costa Rican heterospiline wasps, a hyperdiverse lineage of parasitoid Braconidae, by mapping them to a robust multi-locus molecular phylogeny. We discover a strong positive relationship between the amount of evolutionary change a character undergoes and how broadly useful the characters are in the context of an interactive identification key- e.g., how evenly the character states are distributed among taxa. The empirical finding that fast characters are the most useful for species identification supports the idea that characters designed for taxonomic diagnoses are likely to underperform- or be positively misleading- in phylogenetic analyses.

## Introduction

Systematics as a biological discipline is broadly concerned with two topics: description of biological diversity, and inference of evolutionary history. While these topics have historically involved different theoretical underpinnings and modes of analysis, both rely on the common currency of *characters*. Characters are observable qualities of an organism- typically morphological, molecular, or behavioral- that are quantified to form basic units for all subsequent analyses.

Consider the simple example of a songbird’s beak. The beak’s shape is a character, and it might be described as having several states: long, short, or crossed. We can observe the state of individual birds, marking each beak as either long, short, or crossed, and compiling the data into a matrix. The data can then serve in a variety of studies, especially when combined with observations of additional characters. A taxonomic project, for example, might try to delimit species by searching for clusters of similar beak shapes. A phylogenetic project might use the data in conjunction with models of beak evolution to reconstruct evolutionary histories. In both scenarios the underlying character data is the same.

That the same characters can be employed for both taxonomic and phylogenetic studies does not imply they perform equally in both arenas, however. Systematists have long waged a largely philosophical argument over the supremacy of various character systems (especially, molecular versus morphological data [1–7], and even over the extent to which the efficacy of different characters can be judged, especially *a priori*). Winston [8] provides a clear description of what qualifies a character to be an ideal *diagnostic* (as opposed to *phylogenetic*) character: easily recognizable and tending to be constant within a taxon. Such characters need not reflect basic biological differences. On the other hand, phylogenetically useful characters need not be easy to observe with a specimen in hand, but do need to incorporate the notion of homology. To be maximally useful, character states should not be restricted to a single taxon [9]. Although many systematists recognize that different types of questions merit different types of data, the considerable labor involved in assembling data matrices continues to provide an impetus for conscripting the same data for multiple questions, even if the data were not collected for all possible downstream purposes. What effect might this have on the resulting phylogenetic analyses?

In the present study, we use a robust multi-locus molecular phylogeny of a hyperdiverse clade of braconid wasps as a framework for examining evolutionary patterns of discrete morphological characters developed for species-level taxonomy. The new ability of interactive identification software to quantitatively assess character utility allows us the novel approach of correlating character performance with evolutionary rate. Specifically, we employ the "Best" function in Lucid Player 3.4 [10] to rank characters according to their probability of eliminating taxa when states are selected. As each character is scored for both a utility rank and a measure of evolutionary change, we can provide an empirical estimate of the extent to which evolutionary change influences the usefulness of characters for species identifications.

Our focal organisms are a highly species-rich complex of doryctine braconid wasps associated with the genus *Heterospilus* Haliday. This genus is especially diverse in the Neotropics [11]. Although *Heterospilus* is among the most abundant wasps collected in passive biodiversity surveys in MesoAmerica ( [12], P. Marsh & J. Whitfield pers obs.) the vast majority of species remain undescribed [11] and little has been published about their biology. Where known, species are often ectoparasitic on Coleoptera [13–15], or other holometabolous insects [16] concealed within plant tissue (e.g. twigs, stems, twig nests).

## Methods

### Morphological characters

An ongoing species-level revision of Costa Rican *Heterospilus* provided 47 discrete morphological characters useful for diagnosing species. The characters (File S1) relate to color, sculpture, pilosity, and morphometric proportions, and when employed in conjunction with the interactive identification software Lucid player 3.4 [10], they are sufficient to separate all of the approximately 350 species in the Costa Rican fauna (Marsh & Wild, pers. obs.)

The relative utility of each morphological character was assessed in the framework of Lucid player 3.4 [10]. The Lucid identification process progresses by eliminating taxa as the user selects character states matching those in the specimen to be identified. A central feature to Lucid’s software is a quantitative ranking algorithm- the "Best" function- that automatically directs users at any point in the process to characters with the highest probability of eliminating remaining taxa. In essence, the "Best" characters are those whose character states are most evenly distributed across taxa, such that choosing a state cleaves the candidate taxa into similar sized groups. At any stage of the key, the “Best” function will select the character whose alternate states are most evenly balanced among the remaining taxa, as this maximizes the chance that choosing a character state will eliminate possible identifications. In our experience with *Heterospilus*, a specimen can be identified by selecting as few as four observed character states following the "Best" suggestion. As Lucid’s "Best" algorithm is a quantitative assessment of character utility for taxonomy, we employ it here to rank order all 47 characters from most taxonomically useful (=more even state distribution among taxa) to least useful (=more skewed state distribution among taxa), with the full set of c. 350 species in the matrix.

### Phylogenetic inference

We inferred the relationships of 95 species of doryctine wasps, focusing on Costa Rican *Heterospilus* and related genera, using 4.3kB of sequence data from 5 loci: nuclear protein coding gene fragments from Alpha Spectrin, RNA Polymerase II, and Carbamoyl Phosphate Synthetase (CAD), the nuclear ribosomal gene 28S, and the mitochondrial protein-coding gene *COI*. Specimen data and Genbank accessions are listed in Table 1, and primer sequences are provided in Table 2. This taxon sample is necessarily smaller than the 350 species in the Costa Rican fauna, as most species are known only from older collections with degraded DNA, while some are represented by single specimens. Thus, we limited our phylogenetic sample to all available specimens collected into ethanol within the past 8 years. In addition to Costa Rican collections, we included seven freshly-collected specimens from Ecuador and two from warm temperate North America. Additional doryctine genera were included in the analysis because of potential paraphily of *Heterospilus* [12].

**Table 1:** Genbank Accessions for the 5 markers used to generate the phylogeny.

**specimen**	**Alpha Spectrin**	**CAD**	**RNA Polymerase II**	**COI**	**28S**
Aleiodes sp. ALW-2011 voucher AW091	JN212224.1	JN212497.1	JN212312.1	JN212139.1	JN212401.1
Allorhogas sp. ALW-2011 voucher AW023	JN212225.1	JN212498.1	JN212313.1	JN212140.1	JN212402.1
Allorhogas sp. ALW-2011 voucher AW069	JN212226.1		JN212314.1	JN212141.1	JN212403.1
Allorhogas sp. ALW-2011 voucher AW080	JN212227.1	JN212499.1	JN212315.1		JN212404.1
Allorhogas sp. ALW-2011 voucher AW089	JN212228.1	JN212500.1	JN212316.1	JN212142.1	JN212405.1
Allorhogas sp. ALW-2011 voucher AW097	JN212229.1	JN212501.1	JN212317.1	JN212143.1	JN212406.1
Allorhogas sp. ALW-2011 voucher AW133	JN212230.1	JN212502.1	JN212318.1	JN212144.1	JN212407.1
Allorhogas sp. ALW-2011 voucher AW142	JN212287.1	JN212503.1	JN212319.1	JN212145.1	JN212408.1
Aphelopsia *annulicornis* voucher AW011	JN212235.1	JN212506.1	JN212322.1	JN212148.1	JN212411.1
Barbalhoa sp. ALW-2011 voucher AW050	JN212233.1	JN212507.1	JN212323.1	JN212149.1	JN212412.1
Caenophanes sp. ALW-2011 voucher AW123	JN212234.1		JN212324.1	JN212150.1	JN212413.1
Heterospilus sp. ALW-2011 voucher AW015	JN212247.1	JN212518.1	JN212337.1	JN212162.1	JN212426.1
Heterospilus sp. ALW-2011 voucher AW016	JN212249.1	JN212519.1	JN212338.1	JN212163.1	JN212427.1
Heterospilus sp. ALW-2011 voucher AW021	JN212252.1	JN212520.1	JN212339.1	JN212164.1	JN212428.1
Heterospilus sp. ALW-2011 voucher AW026	JN212254.1	JN212521.1		JN212165.1	JN212429.1
Heterospilus sp. ALW-2011 voucher AW027	JN212255.1	JN212522.1	JN212340.1	JN212166.1	JN212430.1
Heterospilus sp. ALW-2011 voucher AW047	JN212248.1	JN212523.1	JN212341.1	JN212167.1	JN212431.1
Heterospilus sp. ALW-2011 voucher AW048	JN212258.1		JN212342.1	JN212168.1	JN212432.1
Heterospilus sp. ALW-2011 voucher AW052	JN212260.1	JN212524.1	JN212343.1	JN212169.1	JN212433.1
Heterospilus sp. ALW-2011 voucher AW063	JN212240.1	JN212525.1	JN212344.1	JN212170.1	JN212434.1
Heterospilus sp. ALW-2011 voucher AW068	JN212261.1	JN212526.1	JN212345.1		JN212435.1
Heterospilus sp. ALW-2011 voucher AW071	JN212246.1		JN212346.1	JN212171.1	JN212436.1
Heterospilus sp. ALW-2011 voucher AW073	JN212262.1		JN212347.1	JN212172.1	JN212437.1
Heterospilus sp. ALW-2011 voucher AW074	JN212263.1	JN212527.1	JN212348.1	JN212173.1	JN212438.1
Heterospilus sp. ALW-2011 voucher AW077	JN212265.1	JN212528.1	JN212349.1	JN212174.1	JN212439.1
Heterospilus sp. ALW-2011 voucher AW081	JN212266.1		JN212350.1		JN212440.1
Heterospilus sp. ALW-2011 voucher AW082	JN212267.1	JN212529.1	JN212351.1	JN212175.1	JN212441.1
Heterospilus sp. ALW-2011 voucher AW083	JN212268.1	JN212530.1	JN212352.1	JN212176.1	JN212443.1
Heterospilus sp. ALW-2011 voucher AW084	JN212269.1	JN212531.1	JN212353.1		JN212444.1
Heterospilus sp. ALW-2011 voucher AW086			JN212354.1	JN212177.1	JN212445.1
Heterospilus sp. ALW-2011 voucher AW088	JN212270.1	JN212532.1		JN212178.1	JN212446.1
Heterospilus sp. ALW-2011 voucher AW092	JN212271.1	JN212533.1		JN212179.1	JN212447.1
Heterospilus sp. ALW-2011 voucher AW094	JN212272.1	JN212534.1	JN212355.1	JN212180.1	JN212448.1
Heterospilus sp. ALW-2011 voucher AW095	JN212273.1			JN212181.1	JN212449.1
Heterospilus sp. ALW-2011 voucher AW096	JN212274.1	JN212535.1	JN212356.1	JN212182.1	JN212450.1
Heterospilus sp. ALW-2011 voucher AW098			JN212357.1	JN212183.1	JN212451.1
Heterospilus sp. ALW-2011 voucher AW099		JN212536.1	JN212358.1	JN212184.1	JN212452.1
Heterospilus sp. ALW-2011 voucher AW100	JN212275.1	JN212537.1	JN212359.1		JN212453.1
Heterospilus sp. ALW-2011 voucher AW102	JN212276.1	JN212538.1			JN212442.1
Heterospilus sp. ALW-2011 voucher AW103	JN212277.1	JN212539.1	JN212360.1	JN212185.1	JN212454.1
Heterospilus sp. ALW-2011 voucher AW104	JN212278.1		JN212361.1	JN212186.1	JN212455.1
Heterospilus sp. ALW-2011 voucher AW105	JN212279.1	JN212540.1	JN212362.1	JN212187.1	JN212456.1
Heterospilus sp. ALW-2011 voucher AW106		JN212541.1	JN212363.1	JN212188.1	JN212457.1
Heterospilus sp. ALW-2011 voucher AW108			JN212364.1		JN212458.1
Heterospilus sp. ALW-2011 voucher AW111	JN212280.1	JN212542.1	JN212365.1		JN212459.1
Heterospilus sp. ALW-2011 voucher AW112	JN212281.1	JN212543.1	JN212366.1	JN212189.1	JN212460.1
Heterospilus sp. ALW-2011 voucher AW114	JN212244.1	JN212544.1	JN212367.1	JN212190.1	JN212461.1
Heterospilus sp. ALW-2011 voucher AW126	JN212282.1	JN212545.1	JN212368.1	JN212191.1	JN212462.1
Heterospilus sp. ALW-2011 voucher AW132			JN212369.1	JN212192.1	JN212463.1
Heterospilus sp. ALW-2011 voucher AW135	JN212284.1	JN212546.1		JN212193.1	JN212464.1
Heterospilus sp. ALW-2011 voucher AW141	JN212294.1	JN212547.1	JN212370.1	JN212194.1	JN212465.1
Heterospilus sp. ALW-2011 voucher AW147	JN212288.1	JN212548.1		JN212195.1	JN212466.1
Heterospilus sp. ALW-2011 voucher AW149	JN212290.1	JN212549.1	JN212371.1	JN212196.1	JN212467.1
Heterospilus sp. ALW-2011 voucher AW151	JN212291.1	JN212550.1	JN212372.1	JN212197.1	JN212468.1
Heterospilus sp. ALW-2011 voucher AW153	JN212292.1	JN212551.1	JN212373.1	JN212198.1	JN212469.1
Heterospilus sp. GR1 voucher AW075	JN212243.1	JN212512.1	JN212329.1	JN212155.1	JN212418.1
Heterospilus sp. GR10 voucher AW031	JN212241.1	JN212510.1	JN212327.1	JN212153.1	JN212416.1
Heterospilus sp. GR102 voucher AW033	JN212239.1	JN212508.1	JN212325.1	JN212151.1	JN212414.1
Heterospilus sp. GR102 voucher AW148	JN212289.1	JN212509.1	JN212326.1	JN212152.1	JN212415.1
Heterospilus sp. GR19 voucher AW025	JN212242.1	JN212511.1	JN212328.1	JN212154.1	JN212417.1
Heterospilus sp. GR20 voucher AW076	JN212264.1	JN212513.1	JN212330.1	JN212156.1	JN212419.1
Heterospilus sp. GR37 voucher AW137	JN212245.1	JN212514.1	JN212331.1	JN212157.1	JN212420.1
Heterospilus sp. GR62 voucher AW041	JN212257.1	JN212515.1	JN212332.1	JN212158.1	JN212421.1
Heterospilus sp. SM13 voucher AW070			JN212333.1		JN212422.1
Heterospilus sp. SM67 voucher AW049	JN212259.1	JN212516.1	JN212334.1	JN212159.1	JN212423.1
Heterospilus sp. SM84 voucher AW139	JN212286.1		JN212335.1	JN212160.1	JN212424.1
Heterospilus sp. SM97 voucher AW035	JN212256.1	JN212517.1	JN212336.1	JN212161.1	JN212425.1
Heterospilus sp. ST1A voucher AW107	JN212295.1	JN212552.1	JN212374.1	JN212199.1	JN212470.1
Heterospilus sp. ST2 voucher AW024	JN212253.1	JN212554.1	JN212376.1	JN212201.1	JN212472.1
Heterospilus sp. ST2 voucher AW072	JN212293.1	JN212555.1	JN212377.1		JN212473.1
Heterospilus sp. ST28 voucher AW009	JN212299.1	JN212553.1	JN212375.1	JN212200.1	JN212471.1
Heterospilus sp. ST31 voucher AW129	JN212283.1	JN212556.1	JN212378.1	JN212202.1	JN212474.1
Heterospilus sp. ST34 voucher AW140	JN212296.1	JN212557.1	JN212379.1	JN212203.1	JN212475.1
Heterospilus sp. ST36 voucher AW130			JN212380.1	JN212204.1	JN212476.1
Heterospilus sp. ST4 voucher AW046	JN212297.1	JN212559.1	JN212382.1	JN212206.1	JN212478.1
Heterospilus sp. ST44 voucher AW017	JN212250.1	JN212558.1	JN212381.1	JN212205.1	JN212477.1
Heterospilus sp. ST54B voucher AW127	JN212298.1		JN212383.1	JN212207.1	JN212479.1
Heterospilus sp. ST63 voucher AW045	JN212300.1	JN212560.1	JN212384.1	JN212208.1	JN212480.1
Heterospilus sp. ST64A voucher AW136	JN212285.1		JN212385.1	JN212209.1	JN212481.1
Heterospilus sp. ST69C voucher AW019	JN212251.1	JN212561.1	JN212386.1	JN212210.1	JN212482.1
Hypodoryctes sp. ALW-2011 voucher AW122	JN212303.1	JN212565.1	JN212389.1	JN212214.1	JN212486.1
Johnsonius sp. ALW-2011 voucher AW039	JN212301.1	JN212562.1	JN212387.1	JN212211.1	JN212483.1
Johnsonius sp. ALW-2011 voucher AW138	JN212237.1	JN212563.1		JN212212.1	JN212484.1
Leluthia flavocoxalis voucher AW013	JN212302.1	JN212564.1	JN212388.1	JN212213.1	JN212485.1
Notiospathius angustus voucher AW007	JN212304.1	JN212566.1	JN212390.1	JN212215.1	JN212489.1
Notiospathius ornaticornis voucher AW010	JN212306.1	JN212567.1	JN212391.1		JN212490.1
Notiospathius ornaticornis voucher AW014	JN212236.1	JN212568.1	JN212392.1	JN212216.1	JN212488.1
Notiospathius sp. ALW-2011 voucher AW008	JN212305.1	JN212569.1	JN212393.1	JN212217.1	JN212491.1
Notiospathius sp. ALW-2011 voucher AW042	JN212307.1	JN212570.1	JN212394.1	JN212218.1	JN212487.1
Notiospathius sp. ALW-2011 voucher AW144	JN212308.1	JN212571.1	JN212395.1	JN212219.1	JN212492.1
Pambolus sp. ALW-2011 voucher AW125	JN212238.1	JN212572.1		JN212220.1	JN212493.1
Pioscelus sp. ALW-2011 voucher AW012	JN212231.1	JN212504.1	JN212320.1	JN212146.1	JN212409.1
Pioscelus sp. ALW-2011 voucher AW109	JN212232.1	JN212505.1	JN212321.1	JN212147.1	JN212410.1
Spathius calligaster voucher AW150	JN212309.1	JN212573.1	JN212396.1	JN212221.1	JN212494.1
Spathius evansi voucher AW152	JN212310.1	JN212574.1		JN212222.1	JN212495.1
Stiropius sp. ALW-2011 voucher AW004	JN212311.1	JN212575.1	JN212397.1	JN212223.1	JN212496.1

**Table 2:** Primers.

Primer	Locus	Sequence	Direction	Source
AS1794F	Alpha Spectrin	GTGGGTTCNGAYGAYTAYGGTCG	F	Wild & Maddison 2008
AS1822F2	Alpha Spectrin	AGCCACGARCCHGCNATHCAAGC	F	this study
AS2053R	Alpha Spectrin	TCCTCCTCAGCRTTYTCRAACCANGA	R	Wild & Maddison 2008
CD284F	CAD	CAGATACGGTAATCGYGGNCAYAA	F	this study
CD285F	CAD	TACGGTAATCGCGGNCAYAAYCARCC	F	this study
CD688R	CAD	TGTATACCTAGAGGATCDACRTTYTCCATRTTRCA	R	this study
CD684R	CAD	ACGTTCTCCATRTTRCADACNGTGATGCA	R	this study
PL457F	RNA Pol II	CAGCCTACACTACAYAARATGAGTATGATGG	F	this study
PLR1	RNA Pol II	TCAGGACCGTAATCRTCYTTRATRAARTG	R	this study
PLR2	RNA Pol II	GCAAGATACGARTTYTCNACRAANCCYCT	R	this study
CX24F1	COI	TCAGGAATAGTNGGTTTATCWATAAG	F	this study
CX342R	COI	TGAGCAACAACGTAATAWGTATCATG	R	this study
LCO1490	COI	GGTCAACAAATCATAAAGATATTGG	F	Folmer 1994
HCO2198	COI	TAAACTTCAGGGTGACCAAAAAATCA	R	Folmer 1994
D2F	28S	AGAGAGAGTTCAAGAGTACGTG	F	Mardulyn & Whitfield 1999
D3R	28S	TAGTTCACCATCTTTCGGGTCCC	R	Mardulyn & Whitfield 1999

Genomic DNA was non-destructively extracted from the intact mesothorax, metathorax, and mesosoma after removing the head and prothorax. Specimens were soaked for 4-12 hours in a proteinase K solution, and the DNA was isolated using a Qiagen DNeasy kit according to the manufacturer’s protocol. Each sampled specimen was then scored for the Lucid key characters. The morphological matrix was >95% complete, as species sampled only from males and specimens with damaged antennae precluded observation of ovipositor and antennal characters for several taxa. Voucher specimens are deposited in the Illinois Natural History Survey (INHS) collections.

DNA was amplified in a polymerase chain reaction using Takara Ex Taq and the manufacturer’s reagents under the recommended protocol. The nuclear protein-coding genes were amplified using a 2-stage nested PCR (described in [17]), with extension times adjusted to suit the length of the target fragment and annealing temperatures in accordance with primer Tm. PCR product was purified using Qiagen Qiaquick elution columns per the manufacturer’s protocol, and the DNA was sequenced using Sanger sequencing on an ABI 3730XL capillary sequencer. Chromatograms were edited in BioEdit [18] and aligned in Mesquite 2.7 using Opal [19].

Phylogenies were inferred for individual loci and for the concatenated data using MrBayes 3.1 [20], with substitution models selected using MrModeltest [21]. We employed a 5-partition set for the final analyses as follows: 28S, COI codon positions 1 & 2; COI codon position 3; nuclear protein-coding genes codon positions 1 & 2; and nuclear protein-coding genes codon position 3. We replicated the final MrBayes analysis twice, for over 2.5 x10^7^ generations each time, and checked convergence among runs using AWTY [22] and among parameter estimates using Tracer [23]. We obtained an ultrametric tree by reanalyzing the matrix using the same models and partition scheme in BEAST [24] for 5 x 10^7^ generations, using the MrBayes consensus as a starting tree and a relaxed molecular clock model. We did not specify any absolute age constraints.

### Character evolution

Morphological characters were mapped to the ultrametric tree using Mesquite 2.7 [25]. Mesquite scored each character for the number of character state changes in a parsimony framework. These assessments of character change were then plotted against the Lucid "Best" rank, and the statistical significance of the relationship tested with a Spearman Rank two-tailed test.

### Ethics statement

Biological samples used in this study were collected and exported with the requisite permission of the governments of Costa Rica (via INBio) and Ecuador (collection N° 019- IC-FAU-DNBAP/MA and export 011-EXP-CIEN-FAU-DNBAPVS/MA to Lee Dyer). North American samples were collected with the expressed permission of the landowners, no specific permissions were required as the locations are not protected in any way nor did our collections involve endangered or protected species.

## Results & Discussion

### Phylogeny

The concatenated genetic data produced a well resolved tree, with 87% of nodes within the heterospiline clade showing posterior probabilities > 95% and 82% of nodes with a posterior probability of 100%. Topologies generated from individual loci were broadly congruent with each other and with previously published molecular phylogenies [26], differing largely at nodes with low levels of support. MrBayes and BEAST produced topologies identical to each other except for alternate resolutions of two internal nodes, one within *Allorhogas* and the other over the sister-taxon relationship of *Heterospilus* 15 and 71.

**Figure 1:**
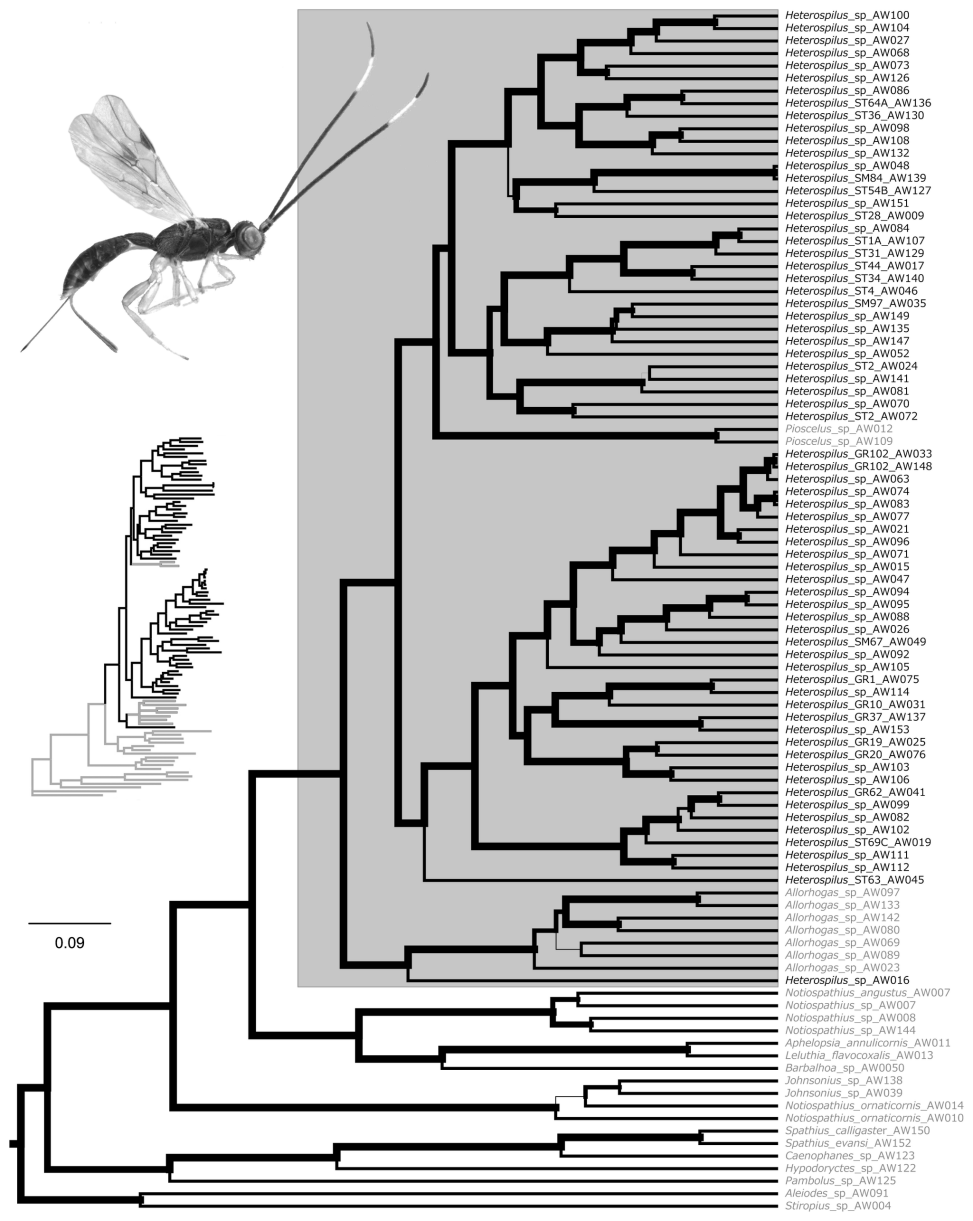
Partitioned Bayesian phylogeny of Costa Rican *Heterospilus* based on 5 loci. The large tree is an ultrametric post-convergence chronogram inferred in BEAST, and the small inset phylogram shows branch lengths from a similar analysis in MrBayes. Edge width represents node posterior probability. *Heterospilus* is inferred to be paraphyletic. All taxa in the shaded rectangle- including the non-*Heterospilus*- were used for subsequent character evolution analyses.

*Heterospilus* was not recovered as monophyletic in the concatenated analysis (Figure 1), nor in analyses of most of the individual loci. The paraphily of *Heterospilus* is not unexpected, as it echoes results from the recent study by Zaldívar-Riverón et al. [12]. In our study, two specimens of *Pioscelus* and seven specimens from the phytophagous genus *Allorhogas* emerged basally within *Heterospilus* with high support. To maintain monophyly of the focal lineage, we coded the relevant characters in *Pioscelus* and *Allorhogas* and included them in the character evolution analysis.

Our small sample of less than 20% of Costa Rican doryctine genera reinforces earlier findings [26,27] that the internal relationships of Doryctinae remain poorly understood. In addition to the paraphily of *Heterospilus*, the Neotropical genus *Notiospathius* consistently emerges in two disparate parts of the outgroup tree. *Notiospathius* is one of several paraphyletic genera in the analyses of Zaldivar-Riverón et al. [26]. The amount of systematic disarray in Doryctinae is perhaps not surprising considering the tremendous and largely undocumented diversity of parasitic Hymenoptera and the small number of taxonomists devoted to the group [28,29].

### Character evolution

**Figure 2:**
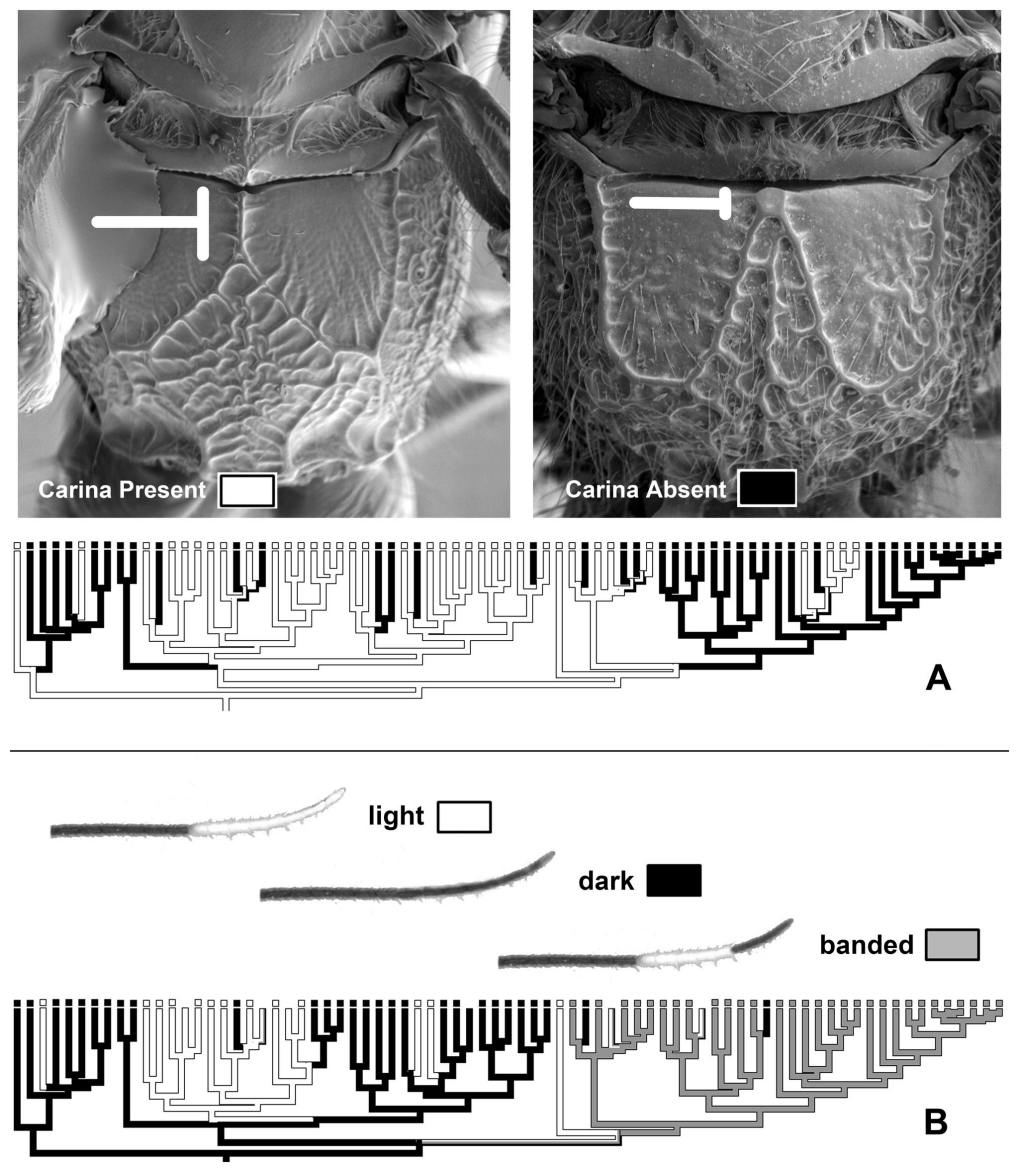
Evolution of 2 of the 47 discrete characters employed in the interactive Lucid key to Costa Rican *Heterospilus*, as inferred in a parsimony framework. A: Presence or absence of the basal median carina on the propodeal dorsum. B: color pattern of the antennal tips (some specimens missing antennae and not scored).

**Figure 3:**
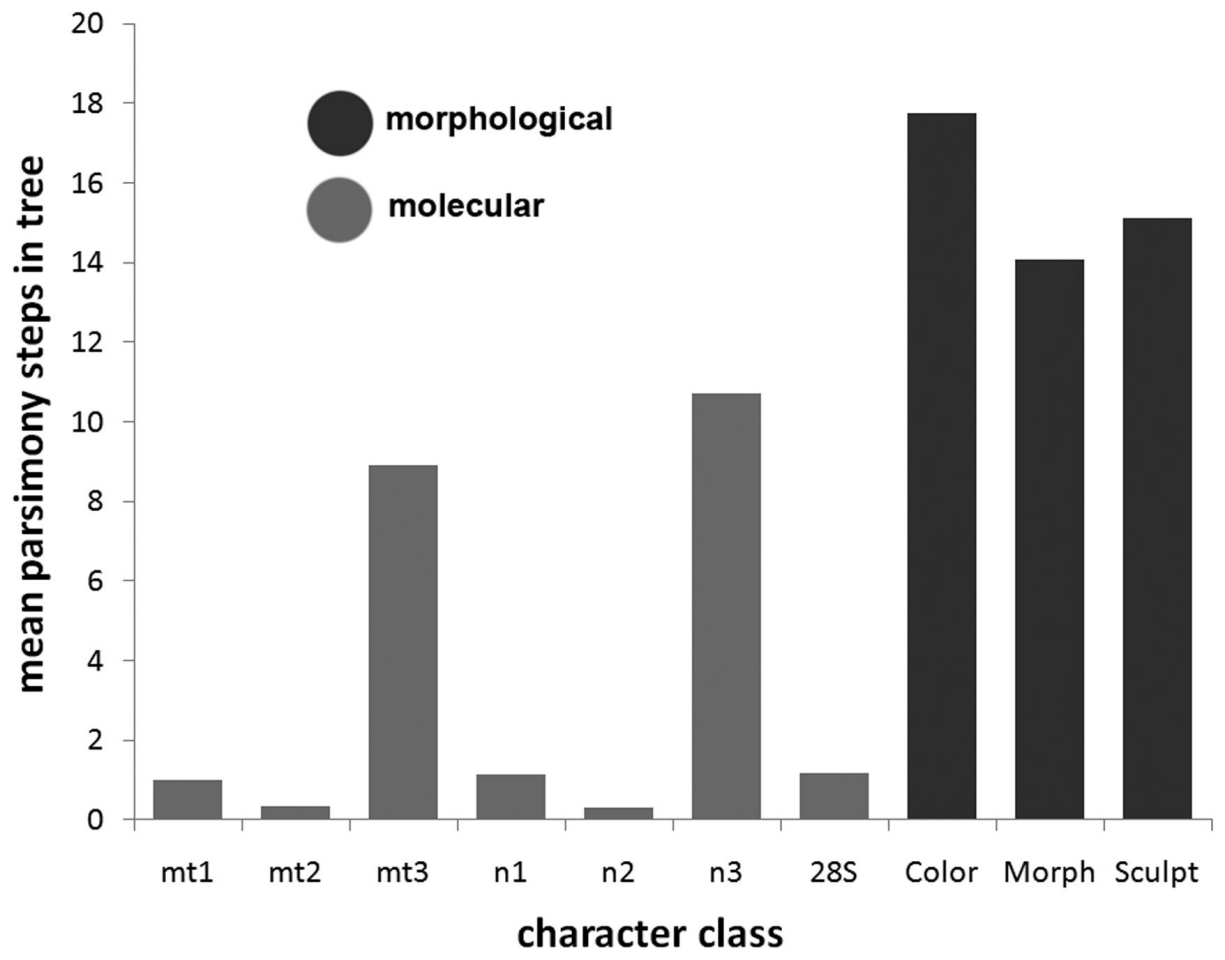
Average number of parsimony steps for different classes of characters. The morphological characters (in black) change state more quickly than even the fastest classes of DNA sequence characters. mt1 = COI first codon position; mt2 = COI second codon position; mt3 = COI third codon position; n1 = combined nuclear genes first codon position; n2 = combined nuclear genes second codon position; n3 = combined nuclear genes third codon position; 28S = 28S nuclear ribosomal gene; Color = combined color characters; Morph = combined morphometric characters; Sculpt = combined sculptural characters.

The vast majority of taxonomic characters (38 of 47) are reconstructed to reverse state at least once (Table 1). Figure 2 illustrates parsimony reconstructions of the evolution of two of these characters. Three characters did not change state at all, reflecting rare states in the full 350-taxon data set that were not picked up in the phylogenetic subsample. The mean number of changes per character inferred on the tree was 15. These rates of evolution are higher than even the 3rd nucleotide positions in the molecular matrix (Figure 3). The high rates of character change and reversals may explain the observed taxonomic confusion among doryctine genera.

**Figure 4:**
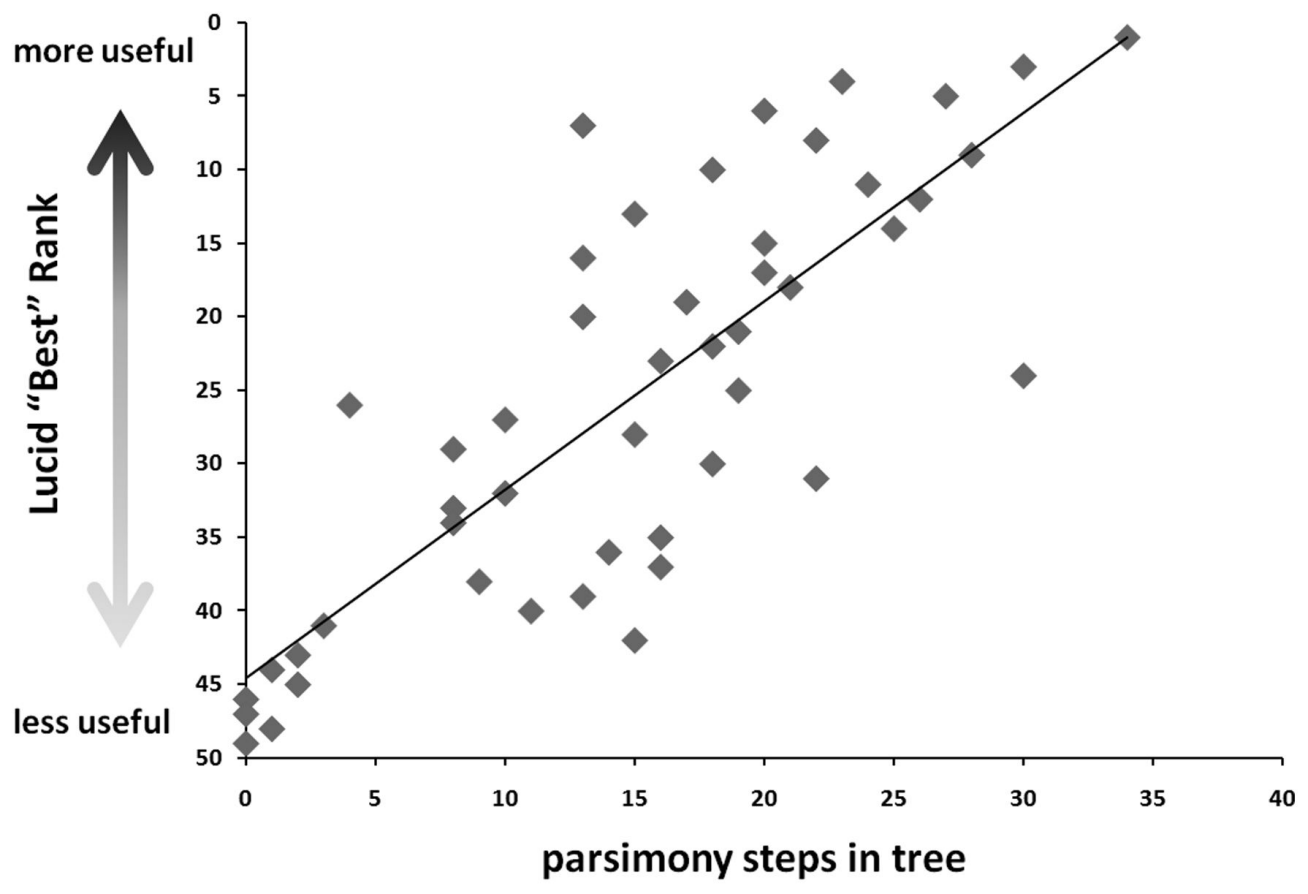
Characters that change state frequently are more likely to be broadly useful for taxonomic diagnosis in an interactive Lucid key (Spearman Rank Correlation; n=47, r = .80, P < .0001).

We found a strong and significant correlation between the Lucid "Best" rank and the number of state changes (Figure 4, Spearman Rank Correlation coefficient = -.80, n = 47, 2-tailed test, P < .0001). This correlation is not an artifact character state number; the relationship holds within characters of the same number of states (2-state characters, Spearman Rank Correlation coefficient = -.76, n = 19, 2-tailed test, P < .0002; 3-state characters, Spearman Rank Correlation coefficient = -.76, n = 17, 2-tailed test, P < .0005; 4-state characters, Spearman Rank Correlation coefficient = -.67, n = 10, 2-tailed test, P < .05; 6 state characters not tested as there were only three). Thus, characters that change state frequently are the most useful for species diagnosis in this group of wasps.

The correlation is possibly due to the phenomenon whereby independent characters that evolve rapidly often find themselves in novel combinations with other characters, providing unique character combinations that allow for easy diagnosis. Thus, fast homoplastic characters may be best for species diagnosis. A logical next step in exploring this phenomenon would be to simulate characters of varying rates on a tree, record the recovered patterns of homoplasy, code the final states in an interactive key, and verify that the artificial characters produce the same correlation of rate to taxonomic utility.

We do not intend these results as commentary on the "molecules v. morphology" debate in phylogenetics, or as a general statement about morphology. Our characters are not a random sample of all possible morphological traits, but were developed specifically because they were useful for taxonomic identification. In fact, morphological characters freed from the demands of phylogenetic inference should encourage taxonomists to be bolder in developing new systems for diagnosis, as long as the characters are recognized as such.

The correlation between evolutionary rate and diagnostic utility illustrates a tension between the properties of characters that render them suitable for taxonomic questions and those that render them suitable for phylogenetic questions. Characters that evolve as quickly as those observed here will saturate in the deeper regions of the tree, providing little useful phylogenetic signal. Consequently, we recommend that data culled for taxonomic projects be used primarily for taxonomy. Although our study does not directly address the converse, it is likely that data collected for phylogenetic projects are likewise best used primarily for phylogenetics. Just because a character matrix exists does not mean it ought be used to answer questions for which it was not designed.

## Supporting Information

File S1 **Morphological characters.**
(DOCX)
