# Supplementary material for: Fast-Evolving Homoplastic Traits Are Best for Species Identification in a Group of Neotropical Wasps
Source: PLoS One. 2013 Sep 11;8(9):e74837. doi: 10.1371/journal.pone.0074837 (PMC3770636; doi:10.1371/journal.pone.0074837)
Supplement: File S1 — Morphological characters. (DOCX) [file pone.0074837.s001.docx]

**Supplemental file 1: Morphological Character Matrix**

Characters:

1 color_of_flagellomeres / white banded dark,

2 base_color_of_scape / brown yellow,

3 stripe_on_scape / present absent,

4 vertex_sculpture / smooth striate granular 'rugose/costate',

5 vertex_color / yellow light_to_medium_brown dark_brown_to_black bicolored,

6 frons_sculpture / smooth striate_or_costate granular,

7 face_sculpture / smooth striate granulate rugose 'rugose-costate' areolate,

8 face_width / '< height' '> height',

9 height_of_malar_space / '> 1/4 eye height' '= 1/4 eye height' '< 1/4 eye height' absent,

10 temple_shape / broad narrow,

11 'ocell-ocular distance' / '1-1.5 x diam lat occ' '1.5-2.5 x diam lat occ' '> 2.5 x diam lat occ',

12 mesosoma_color / yellow light_to_med_brown dark_brown_to_black bicolored,

13 mesopleuron_sculpture / smooth 'costate/striate' 'smooth abv stern, cost/straite dors' granulate,

14 sternaulus_sculpture / scrobiculate smooth,

15 sternaulus_length / '< width of mesopl' '= width of mesopl',

16 mesoscutum_lobe_sculpture / granular smooth 'costate/striate' rugose_along_notauli,

17 mesoscutum_lobe_pilosity / entirely_hairy hairy_only_along_nautali,

18 notauli_sculpture / scrobiculate smooth unsculptured,

19 notauli_junction_with_prescutellar_furrow / triangular_rugose triangular_costate rectangular_rugose unsculptured, 20 scutellum_sculpture / smooth granular rugose,

21 propodeum_basal_median_area_definition / indistinct distinct_and_margined 'distinct, not margined',

22 propodeum_basal_media_area_sculpture / smooth granular rugose,

23 basal_median_carina / distinct absent,

24 areolar_margin / distinct indistinct,

25 areolar_sculpture / areolate 'areolate-rugose' rugose,

26 propodeum_lateral_area_sculpture / entirely_rugose entirely_granulate 'rugose apically, other basally',

27 tubercle_above_hind_coxa_ / pointed not_pointed,

28 apical_lateral_corners / present absent,

29 relative_lenghts_of_veins_r_and_3RSA / 'r < 3RSa' 'r > 3RSa' 'r = 3RSa',

30 'position of vein 1 cu-a' / '1cu-a beyond 1m' '1cu-a interstitial with 1m',

31 1stigma_color / brown bicolored yellow,

32 stigma_width / '< vein r' '= vein r' '> vein r',

33 'hind wing vein SC+R' / present absent,

34 '1relative lengths of veins m+cu and 1m' / '1M+CU > 1M' 'M+CU < 1M' 'M+CU = 1M',

35 leg_color / yellow_or_light_brown bicolored,

36 mesosoma_ventral_sculpture / smooth granular,

37 metasoma_tergum_1_sculpture / costate rugose granulate,

38 metasoma_tergum_2_sculpture / costate granulate smooth,

39 metasoma_tergum_2_width / '4 + x length' '3 - x length',

40 metasoma_tergum_2_color / yellow brown_to_black bicolored,

41 metasoma_tergum_3_sculpture / costate_entirely granulate_entirely granulate_with_smooth_apex costate_with_smooth_apex costate_with_granulate_apex smooth_entirely,

42 'metasoma terga 2+3 ant transverse groove' / 'present, straight' 'present, sinuate' absent,

43 'metasoma terga 2+3 ant post transverse groove' / present absent,

44 'metasoma terga 4-6 sculpture' / smooth costate_at_base granulate 'granulate at base, smooth at apex',

45 'metasoma terga 4-6 color' / concolorous_with_anterior some_or_all_seg_lighter_than_anter,

46 Ovipositor_Length / shorter_than_terga_1 equal_to_terga_1 'equal to terga 1+2' 'equal to 1/2 metasoma' equal_to_metasoma greater_than_metasoma,

47 Temple_width / greater_than_EW equal_to_EW less_than_EW,

48 Terga_1_length / less_than_width equal_to_width greater_than_width,

49 Prescutellar_furrow_carinae / less_than_three three_or_more ;

_______________________________________________________________________

MATRIX

Allorhogas_sp_AW023 2112022101103100100101102201101201010010401202211

Allorhogas_sp_AW069 2112322111133000102111102201100201110012501012221

Allorhogas_sp_AW080 2112022111203000102111102211101211010010301005220

Allorhogas_sp_AW089 0011313101123000102002102211101201110011301015221

Allorhogas_sp_AW097 2112322101113100102111102211100201010001401212001

Allorhogas_sp_AW133 2112023100203100102111102011202201110000401202211

Pioscelus_sp_AW012 2110303111123010100002111201000201010012311013221

Pioscelus_sp_AW109 2110203121023010100002111001000201010011511002220

Heterospilus_GR102_AW033 1102222121133100101121110001000211100011300113211

Heterospilus_GR102_AW063 1102222121133100101121110001000211100011300113221

Heterospilus_GR10_AW031 1102222111123000102121111011000201010011400201201

Heterospilus_GR19_AW025 1012223111123100100120101001000201110011400112211

Heterospilus_GR31_AW075 1012223111123000100121110011000202010011400203201

Heterospilus_GR36_AW114 2012222111213100101121110011200201010011400214101

Heterospilus_GR37_AW137 101(1 2)223121133100100121111001200201010001500001201

Heterospilus_GR42_AW071 1012222101223100101121110011010211010011300011121

Heterospilus_GR87_AW015 11122221(0 1)1123100113111102011000211110001500011210

Heterospilus_GR99_AW047 110220211011310010112111201101121111001230010?0?1

Heterospilus_sp_AW016 2110200101123000100111002011000201010011300002221

Heterospilus_sp_AW017 ?111312111233000100111002211011201010011501013221

Heterospilus_sp_AW019 1101213100220101101010012211001202000011300014201

Heterospilus_sp_AW021 1102222121123000101121111001010211100011300001221

Heterospilus_sp_AW024 0101200111(1 2)23100101111002211000201110002211014220

Heterospilus_sp_AW026 1102222121(1 2)23101113002112001010211000012300011221

Heterospilus_sp_AW027 211(0 1)200101203000101111002011012201010010300002221

Heterospilus_sp_AW035 (0 1)01010010021010110301010201121220110001130001320(0 1)

Heterospilus_sp_AW041 1102324100123000102121112001001201110011300004201

Heterospilus_sp_AW049 1110200101223101113011002011010211110011500004020

Heterospilus_sp_AW052 0110200121123101100010002011001201100012511014220

Heterospilus_sp_AW068 0111100101113000100111002011011211010011300002221

Heterospilus_sp_AW070 0110100111213000101111102211010202010011301002120

Heterospilus_sp_AW073 0101313100222100102122112011001201110011300014101

Heterospilus_sp_AW074 1102222121123100101121110001000211110011300011211

Heterospilus_sp_AW076 1112223101123100101122111001000201010011000113221

Heterospilus_sp_AW077 1102222121123100101121110001000211110011300011211

Heterospilus_sp_AW081 0101212111123100103111002201000201010001110214220

Heterospilus_sp_AW082 1102314101133100100111102201001201010011300012201

Heterospilus_sp_AW083 1102222121123100101121110001000211100011200011221

Heterospilus_sp_AW084 2111310100233101100111002211011201000011111213011

Heterospilus_sp_AW086 2110100121120100100011012011000201010011301015220

Heterospilus_sp_AW088 11022201111231001131110020110002??10001150001?0?0

Heterospilus_sp_AW092 1102222121133100103120112201000211100012300113221

Heterospilus_sp_AW094 1102220110223101113010002011010211000011500010021

Heterospilus_sp_AW095 1102220110123101113010002011010211000011500010021

Heterospilus_sp_AW096 1102222111123100113111112211000211010011300012021

Heterospilus_sp_AW098 2111312110123000103011102011000202010011301003210

Heterospilus_sp_AW099 110030110112310010011100220100120201??????????2?0

Heterospilus_sp_AW100 2010100100220101101011002011202201000011301013211

Heterospilus_sp_AW102 (0 1)101301121123000100111002201001201010011300013221

Heterospilus_sp_AW103 1112220101123100101021111001010201000011300312111

Heterospilus_sp_AW104 ?010200100220000101011002011010201000011300013021

Heterospilus_sp_AW105 1102222111123000103121111011000211010011400214221

Heterospilus_sp_AW106 (0 1)010100110213100103021111011000201000011300113111

Heterospilus_sp_AW108 2110100110210100100011002011000201000011300002200

Heterospilus_sp_AW111 1100300111210001101010002201010212100011300013221

Heterospilus_sp_AW112 2102222101133000102102112001202201110011000112101

Heterospilus_sp_AW126 2111313100123000101111002001000201010011310104220

Heterospilus_sp_AW129 2111314111113000100111002111001201010102111214221

Heterospilus_sp_AW130 2111002121030000101011002011100200000000301003200

Heterospilus_sp_AW132 21103001211200001001220120110002010000(0 1)2300012200

Heterospilus_sp_AW135 21110001002300001011111020110122??10001230001?0?1

Heterospilus_sp_AW136 21113101111200001000110020110002020000(0 1)1300003211

Heterospilus_sp_AW139 2010311100130000102111012010000201100012111215221

Allorhogas_sp_AW142 211202310020101010211101201100020101101000110?021

Heterospilus_sp_AW147 0010300101120101101010002011010201100011521004020

Heterospilus_sp_AW148 1102222121133100101121110001000211100011300112211

Heterospilus_sp_AW149 0101310101123000100122002011010201110011300112221

Heterospilus_sp_AW151 2011313100232002102000111011001201101011301115021

Heterospilus_sp_AW153 1102322111133000100121101011002201110012301114211

Heterospilus_ST2_AW072 0111313100223100102102012211000201110012311014221

Heterospilus_ST30_AW141 ?101311101(1 2)33000101111012201001201000011310114211

Heterospilus_ST31_AW107 2011312101133000100111012211011201010002411313201

Heterospilus_ST34_AW140 0011212121133000102011012011001201000011301313201

Heterospilus_ST4_AW046 0111311100233000100111002211011201110002410204121

Heterospilus_ST54B_AW127 2111311110122000100010012011011201110012400215221

Heterospilus_ST5_AW009 2111312120133000102001112011010201110012100214221

Heterospilus_ST63_AW045 010131012113000010002101201100120110001250001?221
